# Supplementary material for: Catechol-O-methyltransferase and dopamine receptor D4 gene variants: Possible association with substance abuse in Bangladeshi male
Source: PLoS One. 2021 Feb 5;16(2):e0246462. doi: 10.1371/journal.pone.0246462 (PMC7864466; doi:10.1371/journal.pone.0246462)
Supplement: S1 Table — (DOCX) [file pone.0246462.s001.docx]

**S1 Table: Association of COMT polymorphism in Dominant and Recessive model.**

| **Genotype** | **Substance Abuser (n=183)** | **Control (n=175)** | **OR (95% CI)** | ***p*-value** |
| --- | --- | --- | --- | --- |
| **Dominant model** |  |  |  |  |
| GG | 62 | 78 |  |  |
| GA+AA | 121 | 97 | 1.57 (1.027-2.421) | <0.05 |
| **Recessive model** |  |  |  |  |
| GG+GA | 141 | 138 |  |  |
| AA | 42 | 37 | 1.11 (0.682-1.828) | >0.05 |

Fisher's test was performed to calculate statistical significance. p˂ 0.05 was considered as a level of

significance; OR: Odds ratio; CI: Confidence Interval; ns: not significant.
