# Supplementary material for: Catechol-O-methyltransferase and dopamine receptor D4 gene variants: Possible association with substance abuse in Bangladeshi male
Source: PLoS One. 2021 Feb 5;16(2):e0246462. doi: 10.1371/journal.pone.0246462 (PMC7864466; doi:10.1371/journal.pone.0246462)
Supplement: S3 Table — (DOCX) [file pone.0246462.s003.docx]

**S3 Table: Association of the genetic polymorphisms of COMT and DRD4 genes with a period of drug addiction to Methamphetamine and Cannabis abused subjects**

|  | **Genotype** | | **n** | **Age of Onset (years)** | ***p*-value** |
| --- | --- | --- | --- | --- | --- |
| **Methamphetamine**  **(n=78)** | **COMT**  **Val158Met** | Val/Val | 25 | 5.6±0.9 | ns |
|  |  | Val/Met | 39 | 8.5±1.0 |  |
|  |  | Met/Met | 14 | 7.2±1.2 |  |
|  | **DRD4**  **120bp VNTR** | 120 bp/120 bp | 7 | 8.7±1.9 | ns |
|  |  | 120 bp/240 bp | 30 | 7.0±1.0 |  |
|  |  | 240 bp/240 bp | 41 | 7.3±0.9 |  |
| **Cannabis**  **(n=88)** | **COMT**  **Val158Met** | Val/Val | 32 | 9.0±1.1 | ns |
|  |  | Val/Met | 34 | 9.5±0.9 |  |
|  |  | Met/Met | 22 | 9.7±1.4 |  |
|  | **DRD4**  **120bp VNTR** | 120 bp/120 bp | 18 | 8.7±1.4 | ns |
|  |  | 120 bp/240 bp | 32 | 9.0±1.0 |  |
|  |  | 240 bp/240 bp | 38 | 10.0±1.0 |  |

Results were expressed as mean±SEM; Mean age of onset for drug addiction was compared using Analysis of Variance (ANOVA); p<0.05 was considered as a level of significance; ns: not significant.
