# Supplementary material for: Catechol-O-methyltransferase and dopamine receptor D4 gene variants: Possible association with substance abuse in Bangladeshi male
Source: PLoS One. 2021 Feb 5;16(2):e0246462. doi: 10.1371/journal.pone.0246462 (PMC7864466; doi:10.1371/journal.pone.0246462)
Supplement: S4 Table — (DOCX) [file pone.0246462.s004.docx]

**S4 Table: Association of genetic polymorphisms of COMT and DRD4 genes**

**with the age of onset of substance abuse(at different age range)**

|  | **Genotype** | | **n** | **Age of Onset (years)** | ***p*-value** |
| --- | --- | --- | --- | --- | --- |
| **<20**  **(n=120)** | **COMT**  **Val158Met** | Val/Val | 44 | 15.1±0.4 | ns |
|  |  | Val/Met | 46 | 15.9±0.4 |  |
|  |  | Met/Met | 30 | 15.6±0.5 |  |
|  | **DRD4**  **120bp VNTR** | 120 bp/120 bp | 24 | 15.8±0.5 | ns |
|  |  | 120 bp/240 bp | 46 | 15.0±0.5 |  |
|  |  | 240 bp/240 bp | 50 | 15.9±0.4 |  |
| **>20**  **(n=58)** | **COMT**  **Val158Met** | Val/Val | 18 | 27.9±1.2 | ns |
|  |  | Val/Met | 28 | 27.3±1.2 |  |
|  |  | Met/Met | 12 | 25.8±1.2 |  |
|  | **DRD4**  **120bp VNTR** | 120 bp/120 bp | 1 | 22.0±0.0 | ns |
|  |  | 120 bp/240 bp | 22 | 27.7±1.0 |  |
|  |  | 240 bp/240 bp | 35 | 27.0±1.0 |  |

Results were expressed as mean±SEM; Mean age of onset for drug addiction was compared using Analysis of Variance (ANOVA); p<0.05 was considered as a level of significance; ns: not significant.
