# Supplementary material for: Catechol-O-methyltransferase and dopamine receptor D4 gene variants: Possible association with substance abuse in Bangladeshi male
Source: PLoS One. 2021 Feb 5;16(2):e0246462. doi: 10.1371/journal.pone.0246462 (PMC7864466; doi:10.1371/journal.pone.0246462)
Supplement: Attachment — Submitted filename: Reviewer Recommendation and Comments for Manuscript Number PONE-D-20-16794.docx [file pone.0246462.s005.docx]

Comment on Manuscript PONE-D-20-16794: “Catechol-O-methyltransferase and dopamine receptor D4 gene variants: possible association with substance abuse in Bangladeshi male”

With this paper, the authors sought to investigate the association of genetic polymorphism of COMT and DRD4 as a biomarker for increased risk of substance abuse in Bangladesh. To this end used 183 male substance-dependent patients belonging to the age group of 15-49 years and 175 control subjects belonging to the same age group. The authors showed an association between the investigated polymorphisms (COMT and DRD4) with substance abuse, as well as the age of onset, the pattern of drug habit (how many types of substance), and the period of addiction. Also, the study provides interesting information about the relationship between substance use and sociodemographic characteristics. For example, according to the sociodemographic characteristics, a significantly higher percentage of addicted patients were illiterate and unemployed compared to the control subject. These results show that genetic variability may influence the susceptibility to risk of substance abuse as well as addictive characteristics. In an overview, the study provides interesting information on the relationship between substance use and genetic polymorphisms (biological factor), as well as with sociodemographic characteristics for the investigated population. The article too is very well written. However, some information related to ethical care needs to be informed, information about the statistical method needs to be more detailed and the study's findings need to be better discussed for a better understanding of the reader. A couple of issues I would like the authors to give a second thought:

**Abstract:**

Lines 38-40 – Does the information contained in these lines agree with what is presented in the results and the discussion?

**Introduction:** The logic was very well conducted. The authors were able to expose the research problem.

I suggest dividing the first paragraph into two parts.

Line 53 - has more space between factors and [4].

Line 58 - there is an additional parenthesis next to the reference [6].

Line 80 - is missing a space between the receiver and (DRD4).

Line 81 – Put a comma after the quotes.

Line 97 - Put "the" before age.

Lines 62-63 - Is it necessary to explain the mechanism involved in this process or to make it clearer?

**Methods:** It is well detailed about the sample and the genotyping processes, but the statistical procedures still need to be better detailed.

Why did the authors choose to investigate only male individuals?

Was the option due to the greater frequency of substance abuse being male? If yes, provide epidemiological data on this frequency by sex in the introduction. Or was it because of the effect of sex on genotype modulations? Make it clear.

Did participants under the age of 18 sign the free and informed consent form?

Can the quality of the figures be improved? (Figures 1a and 1b)

I consider it extremely important that the authors make the statistical tests clearer (for example, show how the logistic regression was performed) and what the values in the tables represent, mainly about Odds ratios.

**Results:** They are well organized and detailed, but they can be better details or the values in the tables represent, mainly about Odds ratios.

Lines 185-187 - it is written “Here, the frequency of the Val/Val genotype is significantly higher in addicted subjects than control subjects (OR=0.60, 95% CI=0.376-0.957).”, but wouldn't it be the Val/Met genotype? According to the data in table 3. This also does not match the writing of the abstract.

Place table 4 on line 201 and remove from lines 207 and 209.

Table 4 shows the association of the genetic polymorphism of the COMT and DRD4 genes with the risk of specific drug dependence. What was the intention of the authors when presenting the data of the control group? The intention was to categorize dependent individuals into three groups (i.e., users of methamphetamine, heroin, and cannabis) and relate them to genetic polymorphisms. And so, identifying whether there would be a significant effect on a specific type of drug user.

Tables – Standardize mainly on tables 5 and 8.

**Discussion:** I think the authors could have discussed the findings better.

Line 333 – Nucleus accumbens? If yes, I suggest rephrasing the sentence to “mesolimbic dopaminergic centers and dopamine release to the nucleus accumbens”

The authors brought good arguments for the relationship between the COMT polymorphism and the risk of substance abuse, at the end of the second paragraph. However, the arguments for the relationship between DRD4 tandem repeat polymorphism and the risk of substance abuse have been little explored.

What could be the possible explanations for the relationship between the COMT polymorphism and the use of methamphetamine? More specifically, what would be a possible explanation for this finding “the methamphetamine abusers containing heterozygous Val/Met variant showed a significant association with substance abuse compared to Val/Val containing variants”?

Try to provide explanations also for the findings regarding DRD4 tandem repeat polymorphism and cannabis use.

The authors point out some limitations of the study, including the sample being composed only of male individuals. As I said before, I would like to understand why the authors choose only male individuals.

Lines 386-387 – What do the authors mean by developing more rational therapeutic approaches?

In the penultimate paragraph, the authors seek to explain the long duration of addiction in heroin users. However, they do not explain this relationship with the genetic variants of the COMT polymorphism (i.e., Met/Met genotype). Why would a genotype that is associated with a lower activity of the COMT enzyme and consequently a higher amount of dopamine in the synaptic cleft is associated with a longer duration of addiction in heroin users?

The authors could suggest possible future research.
