# Supplementary material for: Catechol-O-methyltransferase and dopamine receptor D4 gene variants: Possible association with substance abuse in Bangladeshi male
Source: PLoS One. 2021 Feb 5;16(2):e0246462. doi: 10.1371/journal.pone.0246462 (PMC7864466; doi:10.1371/journal.pone.0246462)
Supplement: Attachment — Submitted filename: Response to Reviewers.docx [file pone.0246462.s007.docx]

**A point-by-point reply to Reviewers comments:**

**Reviewer # 1:**

**Comment:** In statistical analysis, it is stated that the "odds ratio was measured with a relative risk." Relative risk is a measure for prospective studies, and the odds ratio is a measure for retrospective studies. To clear the confusion, it may be more appropriate to say something like "odds ratio was used as a risk measure."

**Author’s Reply**: Yes, we agree with the reviewer. In statistical analysis, the statement “odds ratio was measured with a relative risk at 95% confidence intervals” was replaced by “odds ratio was used as a risk measure at 95% confidence intervals” (Line: 155).

**Comment:** Also, when I checked the frequencies of tables, just for one comparison, a table has an expected count less than 5, so instead of using Fisher's exact test for all tables, it would be better to use continuity corrected chi-square test if all cells have an expected count higher than 5 and also have an observed count less than 25. If all cells have an expected count higher than 5, and also all cells have an observed count higher than 25, it would be appropriate to use Pearson's chi-square test for determining the significance.

**Author’s Reply:** Authors agreed with the reviewers about this statistical procedure. As the Fischer exacts test is a much more acceptable test for genetic polymorphism analysis by most scientists, authors also used that test. However, upon receiving the reviewer’s comments, the authors reanalyzed the data as recommended and found similar associations as with Fischer Exact tests. The authors updated the information in the manuscript and modified the methodology.

**Comment:** Another issue was the name of the post-hoc procedure used after one-way ANOVA for pairwise comparisons. Please mention its name in the statistical analysis section.

**Author’s Reply:** The post-hoc analysis used after one-way ANOVA was Tukey's Test for post-hoc analysis, which is mentioned in the revised manuscript (Lines: 159-160).

**Comment:** Authors stated that they corrected the footnote of Table 1. But instead of SEM, it still remains as SD.

**Author’s Reply:** Thank you for indicating this mistake. Accordingly, the footnote of Table 1 was corrected from SD to SEM (Line: 183).

**Comment:** As the odds ratios are corrected, at lines 215-217, the statement should also be corrected, instead of saying the heterozygous and 240bp/240bp homozygous are associated with risk of substance abuse, it would be correct to say these two genotypes are protective for substance abuse. Because their odds ratios are lower than 1. This means the reference homozygous 120/120 is more likely to become a substance abuser.

**Author’s Reply:** Yes, we agree with the reviewer. As the odds ratios are lower than 1, in the revised manuscript, the statement “both the heterozygous and homozygous derived variants were found significantly associated with risk of substance abuse compared to the control subjects” was replaced by “Both heterozygous (120bp/240bp) and homozygous (240 bp/240 bp) derived variants were found to show a protective role for substance abuse compared to the control subjects which was statistically significant" (Lines: 217-218).

**Comment:** At lines 232-233, the statement should be heterozygous and 240bp/240bp homozygous have less risk to become a cannabis abuser (odds ratios are less than 1), 120bp/120bp homozygous is more likely to become cannabis abuser.

**Author’s Reply:** According to the suggestion of the reviewer, as the odds ratios are less than 1, the statement “In the case of DRD4 polymorphism, both the heterozygous and homozygous derived variants of cannabis abusers were significantly associated with the risk of cannabis abuse (p<0.01)" was replaced by "In the case of DRD4 polymorphism, it was found that both the heterozygous(120bp/240bp) and homozygous(240bp/240bp) derived variants have less risk to become cannabis abusers which were statistically significant (p<0.01)" in the revised manuscript (Lines: 233- 235)

**Comment:** At lines 257-259, it is stated that post-hoc analysis was done for COMT, as seen in table 5, one-way analysis resulted as ns. If the omnibus test is not significant no need to perform a post-hoc analysis. Also, the statement in the same manner at lines 318-319 is unnecessary as the omnibus test result is p>0.05.

**Author’s Reply:** Very Good Suggestion. In the revised manuscript, the statement "To compare pairwise differences among the age of onset of three different genotypic groups of COMT polymorphism post-hoc analysis was made, no significant difference was found” was deleted accordingly. Also, the statement “Further, after post-hoc analysis, there was no pairwise difference among these genotypic groups of DRD4 polymorphism” at line 318-319 was also deleted accordingly.

**Reviewer #2:**

**Comment:** Line 53 – “very high percentage (93%0 of addicted subjects in Bangladesh are male”. Remove 0 after 93% and close the parenthesis.

**Author’s Reply:** In the revised manuscript at line 53, 0 is removed after 93%, and the parenthesis was closed.

**Comment:** Line 166 – “are less educated (18% illiterate)”. Where is this information found in Table 1? Better to add this information to the table or change it to secondary educated.

**Author’s Reply:** Thank you for indicating the mistake. During the previous revision of the manuscript, due to the unavailability of illiterate subjects in the control group and multivariate analysis, we combined drug-addicted illiterate subjects (18%) with secondary educated subjects and compared them with the graduate subjects. After combining the percentage of secondary educated subjects became 84.7%. By mistake, the information was not changed at line 166. In the revised manuscript, this information is corrected in line 166.

**Comment:** Lines 167-169 – “case of COMT polymorphism, the graduate and employed subjects are less likely to be addicted than the secondary educated and unemployed subjects.” Was that information supposed to appear in the table? The question is being raised, as there is no information about the polymorphism of COMT.

**Author’s Reply:** We performed multiple logistic regression using all the variables and found that in the case of COMT polymorphism, the graduate and employed subjects are less likely to be addicted than the secondary educated and unemployed subjects. We did not mention this association in Table 1, as we have shown all the genetic associations in individual Tables.

**Comment:** Line 191 – “suicidal attempt (12.4%)”. Is this information in accordance with table 2.

**Author’s Reply:** As according to the study, 5.9% of addicted subjects made a suicidal attempt; in the revised manuscript, the information "suicidal attempt (12.4%)" was changed into "suicidal attempt (5.9%)" according to Table 2.

**Comment:** Line 205 – “COMT 158Val/Met” - COMT Val158Met.

**Author’s Reply:** According to the reviewer’s suggestion, “COMT 158Val/Met” is rewritten as “COMT Val158Met” at line 205.

**Comment:** Line 228 - (e.g methamphetamine) => (e.g., methamphetamine)

**Author’s Reply:** According to the reviewer’s suggestion, (e.g. methamphetamine) is rewritten (e.g., methamphetamine) at line 229 in the revised manuscript.

**Comment:** Lines 229-230 - (e.g methamphetamine abuser) => (e.g., methamphetamine abuser)

**Author’s Reply:** According to the reviewer’s suggestion, (e.g methamphetamine abuser) is rewritten (e.g., methamphetamine abuser) at lines 230-231 in the revised manuscript.

**Comment:** Line 335 – “addicted patients were illiterate and unemployed compared to the control subjects” – In line 335, the authors state that a significantly higher percentage of dependent patients was illiterate and point out that this information is present in Table 1, however the information cited is not included.

**Author’s Reply:** As the information about Educational status given in Table 1 did not include illiterate subjects, in the revised manuscript, the statement "a significantly higher percentage of dependent patients was illiterate" was replaced by "a significantly higher percentage of addicted patients were less educated” (Line 334), as in Table 1 it is mentioned that 84.7% addicted subjects had secondary education.

**Comment:** Lines 339-340 – “and the study of Hasam and Mushahid [31] and Islam et al. [32]” => and the studies of Hasam and Mushahid [31] and Islam et al.

**Author’s Reply:** According to the reviewer’s suggestion, “and the study of Hasam and Mushahid [31] and Islam et al. [32]” is rewritten as “and the studies of Hasam and Mushahid [31] and Islam et al.”

**Comment:** Line 345 – “Although only 9.4% abusers” – In the results and in table 2, the value is 9.5%.

**Author’s Reply:** Yes, we agree with the reviewer. According to Table 2, the percentage of addicted subjects with relapse behavior was 9.5%; at line 344 in the revised manuscript, this information is rewritten as “although only 9.5% abusers”.

**Comment:** Lines 348-349 – “significant relationship was found between the heterozygous genotype of COMT polymorphism (Table 3)”. It could indicate the relationship with what, for example, “significant relationship was found between the heterozygous genotype of COMT polymorphism and substance abuse (Table 3)”.

**Author’s Reply:** According to the suggestion of the reviewer, the statement at lines 347-348 in the revised manuscript is rewritten as “a significant relationship was found between the heterozygous genotype of COMT polymorphism and substance abuse."

**Comment:** Lines 362-371 – The authors state that “significant relationship was found between both the derived genotype of DRD4 tandem duplication and risk for substance abuse where according to the odds ratio, both heterozygous (120bp/240bp) (OR=0.37, 95% CI=0.172-0.826) and homozygous (240 bp/240 bp) (OR=0.43, 95% CI=0.193-0.937) derived variants showed association with decreased risk of substance abuse” and “the longer allele (240bp/240bp) of the tandem duplication played a protective role in substance abuse, and according to a previous study by Kereszturi et al., 2007, the shorter allele (120bp/120bp) is found to be the risk allele in novelty seeking and other neuropsychiatric behavior". Justifying why the longer allele is not associated with substance abuse. However, on lines 371-376, the explanation seems to me to be about how the longer allele is associated with substance abuse.

**Author’s Reply:** Yes, we agree with the reviewer that the explanation given in lines 371-376 did not indicate how the longer allele is associated with less substance abuse risk. As the longer allele of DRD4 120bp tandem duplication played a protective role in Bangladeshi substance abused subjects, a better explanation behind the reason is given at lines 370-376 in the revised manuscript.

**Comment:** Line 370 – “by Kereszturi et al., 2007” – standardize citations.

**Author’s Reply:** According to the reviewer's suggestion, the citation is standardized in the revised manuscript (Line 369).

**Comment:** The authors could write a conclusion for the article.

**Author’s Reply:** According to the reviewer's suggestion, a conclusion is added in the revised manuscript (Lines: 448-450).

************
